# Mortality during In-Hospital Treatment for Head and Neck Cancer in Germany: A Diagnosis-Related Group-Based Nationwide Analysis, 2005–2018

**DOI:** 10.1155/2022/1387860

**Published:** 2022-09-17

**Authors:** Isabel Hermanns, Mussab Kouka, Peter Schlattmann, Orlando Guntinas-Lichius

**Affiliations:** ^1^Department of Otorhinolaryngology, Jena University Hospital, Jena, Germany; ^2^Department of Medical Statistics, Computer Sciences and Data Sciences, Jena University Hospital, Jena, Germany

## Abstract

**Background:**

Data on in-hospital MR (IHMR) of head and neck cancer (HNC) are sparse.

**Methods:**

IHMR was determined in Germany between 2005 and 2018 using nationwide population-based diagnosis-related group (DRG) data of 1,090,596 HNC.

**Results:**

The overall average IHMR was 0.04 ± 0.02. IHMR increased with older age to 0.04 ± 0.01 for patients of 65-79 years of age (relative risk [RR] in relation to patients of 35-49 years of age = 1.767; 95%confidence interval [CI] = 1.040 to3.001) to a maximum of 0.07 ± 0.01 for patients of 80 years and older (RR = 2.826; CI = 1.663 to 4.803). IHMR was the highest when no HNC-specific treatment, i.e., best supportive and palliative care, was applied (0.11 ± 0.01; RR in relation to tumor biopsy surgery = 7.241; CI = 3.447 to 5.211). IHMR was not different between surgery, radiotherapy, or chemotherapy/biologicals.

**Conclusions:**

IHMR did not change over time. Efforts are needed to decrease the IHMR for HNC.

## 1. Introduction

Head and neck cancer (HNC) is a frequent and aggressive type of cancer with poor prognosis in advanced stage [1]. More than 300,000 deaths worldwide were estimated due to HNC in 2020 [2]. In Germany, about 9,000 deaths due to HNC were registered for 2020 (database query at the Centre for Cancer Registry Data at the Robert Koch Institute, 31-January-2022; https://http://www.krebsdaten.de/). The overall average 5-year overall survival rate in stages I-II is less than 70% and for stages III-IV less than 40% [3]. Nearly all deaths occur after primary treatment or under palliative care of recurrent/metastatic disease. The issue of early death (within six months of diagnosis) among patients with HNC remains poorly explored [4]. The early death rate is about 9-10% and may be either tumor-related, patient-related, or treatment-related including perioperative mortality [5]. The early death rate has to be differentiated from the in-hospital mortality defined as mortality that occurred during hospitalization. In-hospital mortality can occur within six months of diagnosis and is then congruent to early mortality. Long-term treatment-related complications, tumor recurrence, or cancer-progression related symptoms can also lead to readmission and in-hospital death [6]. In such cases, in-hospital mortality might be not congruent with the early death rate. In-hospital mortality can be an interesting indicator of clinical quality, but there are so far no commonly accepted quality metrics. As a basis, more data on in-hospital mortality for HNC are needed.

We are aware of only a few epidemiologic population-based studies on early mortality or on in-hospital mortality. A recent cross-sectional analysis of United States hospital discharge data from the National (Nationwide) Inpatient Sample (NIS) database including 85,440 patients from 2008 to 2013 showed an in-hospital mortality rate of 4.2% [7]. The NIS was also used to analyze the association of mental health disorders with in-hospital and mortality in head and neck cancer surgery [8]. In the NIS data, mental health disorders were not associated to higher risk of early mortality in patients who underwent surgery from 2003 to 2014 for HNC. Due to the data of the Danish Head and Neck Cancer Group (DAHANCA) registering information on patients with HNC of the oral cavity, pharynx, and larynx, the early mortality rate was 7.1% for all patients treated with curative-intent radio/chemotherapy in Denmark between 2000 and 2017 [9].

Since 2004, German hospitals have to submit diagnosis-related groups (DRG) coding to the insurance company of the patients to receive the reimbursement of hospital stays. In addition, the hospitals annually submit all hospitalization data to the Hospital Remuneration System (InEK) for a continual adjustment of the DRG system. The data are anonymized and also forwarded to the Federal Bureau of Statistics (DESTATIS; https://www.destatis.de/). The DRG data evaluated by the Federal Statistical Office includes various patient-related variables, treatment courses, and in-hospital death of all inpatients who were discharged in virtually all German hospitals during the reporting year and can be used for scientific analyses. For instance, this data source was used to analyze the nationwide in-hospital mortality following colonic cancer resection from 2012 to 2015 [10].

We used the DRG data from 2005 to 2018 to analyze nationwide in-hospital mortality rates for HNC in Germany with focus on the influence of gender, age, tumor localization, treatment types, and trends over the years.

## 2. Material and Methods

The same DRG resources were used as previously reported for the analysis of trends in treatment of head and neck cancer in Germany [11]. The hospitalizations in Germany for the years 2005–2018 were analyzed. Ethics approval was not needed from the local ethics committee. The authors used anonymized data supplied by the German Federal Bureau of Statistics (DESTATIS). The anonymization of such data is regulated in § 16 Bundesstatistikgesetz (German Federal Statistics Act). All authors had access to the study data and reviewed and approved this study.

### 2.1. Patient Cohort Definition

The hospitalizations with a primary diagnosis for head and neck cancer of the International Classification of Diseases, 10th Edition, German Modification (ICD-10-GM), were analyzed: C01-C06 (oral cavity), C07-C13 (salivary glands, nasopharynx, oropharynx, hypopharynx), and C30-C32 (era, nose, paranasal sinus, larynx). Patients with lip cancer (C00) and thyroid cancer (C73) were not included. Patients with skin cancer in the head and neck region were excluded. To compare patients who died in the hospital from others who did not die, two different data queries were programmed: in the first, only cases whose inpatient stay ended with death (coded by the DRG parameter: “reason for discharge” was included. In the second, all cases without the coding for death were included. Subsequently, all cases were grouped according to the OPS procedures (Operationen und Prozedurenschlüssel; OPS, version 2005 to 2018): 1 − 41 = biopsy without incision of the eye, ear, nose and skin of the face, head, and neck; 1 − 42 = biopsy without incision of the mouth, oral cavity, larynx, pharynx, and blood-forming organs; 1 − 43 = biopsy without incision of respiratory organs; 1 − 53 = biopsy through incision on the ear and nose; 1 − 54 = biopsy through incision of the mouth, oral cavity, and pharynx; 5 − 21 = nasal surgery; 5 − 22 = paranasal sinus surgery; 5 − 25 = tongue surgery; 5 − 26 = salivary glands and salivary gland duct surgery; 5 − 27 = other mouth and face surgery; 5 − 28 = surgery in the area of the nasopharynx and oropharynx; 5 − 29 = pharyngeal surgery; 5 − 30 = excision and resection of the larynx; 5 − 301 = hemilaryngectomy; 5 − 31 = other laryngeal and tracheal surgeries; 5 − 401.0 = excision of individual cervical lymph nodes and lymph vessels, including removal of several sentinel lymph nodes; 5 − 403 = radical cervical lymphadenectomy (neck dissection); 8 − 52 = radiation therapy; 8-54 = chemotherapy, immunotherapy, and antiretroviral therapy (hereinafter: chemotherapy/antibody therapy). In a next data query, the variable gender (male, female) was included in the query. In the last step, the variable age cohorts (35 to 49 years, 50 to 64 years, 65 to 79 years, 80+ years) were included in the query. The age cohort from 0 to 34 years was excluded, because too many subgroups with <3 patients were generated. Due to the confidentiality statutes, it is by law not allowed to issue such small subgroups. Therefore, we had to exclude patients with 0 to 34 years of age. The data query for the comparison of the different treatment options included all the head and neck cancer patients without specific treatment of the cancers (i.e., best supportive and palliative care) as covered by the OPS codes described above. The different generation of too small subgroups explains that the variability of the total number of cases finally included in the different data sets. Clinical variables (e.g., TNM classification and tumor histology) were not available, as these are not billing-relevant.

### 2.2. Statistical Analysis

The mortality rate was calculated as the absolute number of all in-hospital deaths in relation to all in-hospital cases. Descriptive statistical analyses were performed using IBM SPSS version 26.0 statistical software for Windows (Chicago, Illinois, USA). Mean values ± standard deviation are presented if not otherwise indicated. All time trend calculations were carried out using SAS version 9.4 (SAS Institute, Cary, North Carolina, USA). Negative binomial regression models with log link were performed to conduct an analysis over time. Here, the dependent variable was the number of cases, and the logarithm of the population at risk was taken as an offset. Three different models were calculation: (a) for age and gender, (b) tumor localization, and (c) tumor treatment. The year was used as covariate. For the regression model on the different primary tumor localization, the patients were grouped into oral cavity cancer, oropharyngeal cancer, laryngeal cancer, hypopharyngeal cancer, and salivary gland cancer. The other subsamples of localizations were too small to be included into this model. Relative risks (RR) with 95% confidence intervals (CI) are reported. For all statistical tests, significance was two-sided and set to *p* < 0.05.

## 3. Results

In total, the data set on in-patient treatment for patients with HNC in German hospitals between 2005 and 2018 contained 1,090,596 cases (78% male; 47% 50-64 years of age; 43,440 deaths, mortality rate: 0,039). The data set on the different tumor localizations contained 1,196,770 cases (28% oral cavity cancer; 3% nasopharyngeal cancer; 16% oropharyngeal cancer; 26% laryngeal cancer; 16% hypopharyngeal cancer; 11%; salivary gland cancer; 44,943 deaths, mortality rate: 0,037). The third data set including all types of therapy had 1,445,033 cases (14% biopsy; 16% chemotherapy/antibody therapy; 26% primary tumor surgery; 10% neck dissection; 16% radiotherapy; 20% no specific primary tumor treatment; 49,635 deaths, mortality rate: 0,034).

### 3.1. Average In-Hospital Mortality Rates for Head and Neck Cancer in Germany

**Figure 1:**
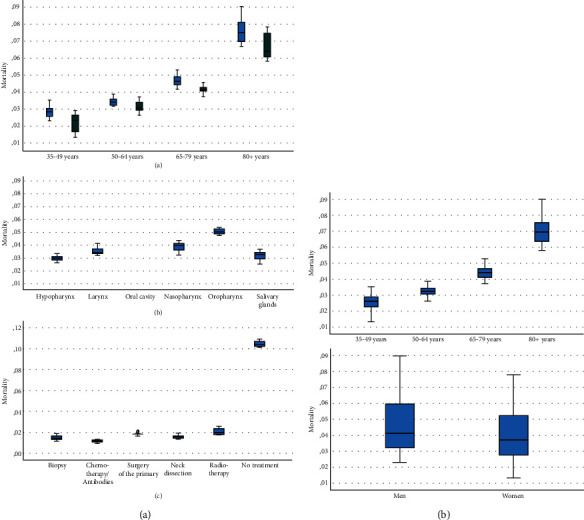
Boxplots on average in-hospital mortality for head and neck cancer in Germany from 2005 to 2018. (a) Age and gender. (b) Tumor localization. (c) Therapy modalities.

Figure 1 and Table 1 give an overview about the average mortality rates for 2005 to 2018, separately for the association to age and gender, tumor localization, and treatment. The overall average mortality rate was 0.0434 ± 0.0183. The mortality rates increased significantly between all four age cohorts (all *p* < 0.001) from 0.0254 ± 0.0057 in the cohort of the patients with 35-49 years of age to a maximum of 0.0710 ± 0.0085 in the cohort of patients 80 years and older. From the perspective of the tumor localization, the average mortality rate was the highest for the oropharynx (0.0512 ± 0.003) and lowest for the hypopharynx (0.0304 ± 0.0025). Concerning the treatment strategies, the mortality rate was the highest (0.1047 ± 0.0026), when no treatment of the HNC was applied and lowest for chemotherapy/antibody therapy (0.0118 ± 0.0012).

### 3.2. Changes of the In-Hospital Mortality Rates between 2005 and 2018

**Figure 2:**
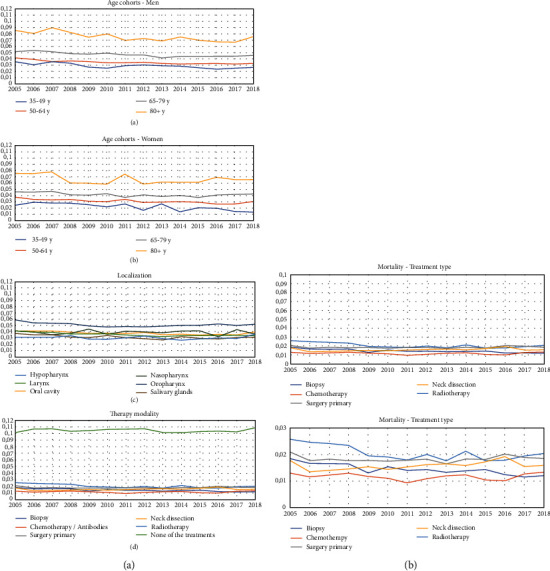
Changes of the annual mortality over time from 2005 to 2018. (a) Age cohorts in men. (b) Age cohorts in women. (c) Tumor localization. (d) Therapy modalities. y: years.

Figure 2 shows the development of the mortality rates over time and separately for age and gender, tumor localization, and treatment. Compared to the patients at 35 to 49 years of age, the relative risk (RR) of in-hospital mortality increased for patients at 65-79 years of age (RR = 1.767; 95%confidence interval [CI] = 1.040 to3.001; *p* = 0.034) and for patients of 80 years and older (RR = 2.826; CI = 1.663 to 4.803; *p* < 0.0001; Table 2). A gender effect was not seen. Related to age and gender, the mortality did not change over time. The tumor localization had no significant influence on the mortality and did not change over time (Table 3). No specific tumor treatment was the only treatment type with increased RR of the mortality compared to biopsy as treatment (RR = 7.241; CI = 3.447 to 15.211; *p* < 0.001; Table 4). The mortality did not change over time for any treatment type.

## 4. Discussion

This study offers for the first time a nationwide perspective on in-hospital mortality of German HNC patients. The average in-hospital mortality rate was 4.0%. We are not aware of any other nationwide study in any other country. The largest publicly available all-payer inpatient care database in the United States, the National Inpatient Sample (NIS) database, is not nationwide but contains data on more than seven million hospital stays. Due to NIS data, the in-hospital mortality between 2000 through 2003 and 2008 through 2013 among hospitalized patients with HNC in the United States was 5.2% and 4.2%, respectively [7, 12]. This is in the same range as the German data and showed a decline overtime, as in the German population could be deduced. One should take into account that some of the patients already die in the emergency departments and during the admission process [13]. These patients do not account to the in-hospital mortality.

Perioperative mortality is part of the in-hospital mortality. 26% of the patients had primary tumor surgery (±10% neck dissection) in the present study. Hence, in these patients, the in-hospital mortality can be equated with perioperative mortality, i.e., on average perioperative mortality that was 1.8% for primary surgery and 1.6% after neck dissection. Also, population-based perioperative mortality data is sparse: the mortality rate after major HNC surgery in England between 2006 and 2011 was higher with 3.0%, but this can be explained by the focus on major surgery [14]. The present study did not differentiate between major and minor surgery. In contrast, the postoperative mortality in a U.S. American NIS series from 2003 to 2014 was <1%; although, the patients' and tumor characteristics look similar to the present study. The differences cannot be explained out of the data.

As no other DRG-based analysis on HNC was performed before, the presented results could only be compared to publications related to other cancers. Due to DRG data, the in-hospital mortality for colon and rectal cancer resection was 5.8% and 3.9%, respectively, from 2012 to 2015 [10, 15]. In-house mortality ranged from 6.2% for gastric resections to 8.1% for pancreatic resections between 2009 and 2017 [16]. For this types of cancer, but also in general for inpatient treatment in Germany, treatment in high-volume hospitals has a lower mortality than very low volume hospitals [17]. Case volume should be analyzed for HNC patients in future studies, too.

Concerning the detected factors influencing the mortality, mainly literature on perioperative mortality and 30-day mortality has to be consulted knowing that 30-day mortality is not congruent to in-hospital mortality: older age and male gender are well known factors for higher early mortality [4, 18]. In the present study, only age but not gender had influence on the in-hospital mortality. If the subsite has an independent influence that is unclear, the results are controversial [4, 19]. In the present study, a specific HNC treatment (surgery, radiotherapy, chemotherapy/biologicals) showed no mortality difference. In a comparable period, the 30-day mortality after radio(chemo)therapy in Denmark was 3.1% [9]. At least in Germany, a potential bias might be included: patients with higher comorbidity or needing adjuvant chemotherapy are rather selected for in-hospital treatment. These factors go along with higher mortality.

Although not much population-based data have been published on time trends, overall, early mortality seems to have decreased over the past decades, at least the 30-day mortality after HNC surgery [20]. The present study did not reveal any mortality change over time neither related to gender, age, tumor localization nor for the treatment type. We would have expected at least a decline for chemotherapy/antibody treatment: there was a substantial shift from classical chemotherapy to cetuximab (introduced 2006) in patients with higher comorbidity [11]. More treatment with cetuximab instead of classical chemotherapy should have been followed by less mortality, but this subgroup of patients might have been too low to have a significant effect. The first checkpoint inhibitor as new treatment option was licensed in 2017 [21]. Hence, these new drugs did not yet play a role in the herein examined period from 2005 to 2018. A decline of mortality could be expected with increased use of immuno-oncology drugs.

Although DRG data is collected prospectively through the routine hospital coding process which collects data from virtually all German hospitals, this study has several limitations. First, the reasons for death remain unclear. The DRG data do not allow a linkage to reasons. HNC-specific reasons can be rupture of large vessels in advanced disease. Cardiovascular or respiratory failure is other frequent reasons, mainly attributed to the severe comorbidity of the patients [22]. Second, this study was retrospective, which could lead to misclassification error and unmeasured variables. Many clinical but important factors with influence on the mortality like higher stage and high comorbidity were not included. The DRG data does indirectly allow a more detailed analysis of the comorbidity and cancer/treatment specific complications by analysis of ICD code grouping for comorbidity [10, 23, 24]. Such an analysis should be performed as a next step to better understand the reasons for in-hospital mortality.

Other factors like nonelective admission, admission at the weekend, or admission to a nonteaching hospital can also have influence on higher in-hospital mortality [7]. Furthermore, a relevant amount of dying HNC patients did not receive a specific HNC treatment. This suggested that these patients may have admitted to the hospital for palliative care during last days of life [25]. HNC patients often live isolated which can make palliative care at home difficult [26]. Vice versa, some patients might have been dismissed from the hospital to allow to die at home [27]. A decrease in mortality over time should have been expected as a sign for better palliative care for HNC patients at home. The DRG data does not allow such an analysis. The present study with unchanged mortality rated over time does not support such a hypothesis. Another aspect has to be emphasized. For patients with curative treatment, the aim should be to decrease the in-hospital mortality. The present data are intended to provide a basis for further research. In contrast, for HNC patients under palliative care, further research is needed why the patients are treated as inpatients and what has to be done that they profit from ambulatory palliative care in the future.

The calculated mortality rates can only be seen as a very good proxy for the true in-hospital mortality rates [28]. The DRG data is primarily collected for reimbursement. Although the data underwent plausibility checks for wrong codes before release of DESTATIS to researchers, it cannot be excluded that the coding followed in some cases more the interest of maximizing the profit than a proper documentation of the actual treatment [29]. As the complete data set is subject to this uncontrolled bias, the comparisons between subsets, like the comparisons between men and women or between subsites, are evenly affected by such a bias.

## 5. Conclusions

The German and nationwide DRG statistics provide a unique epidemiological data source for the quantification of in-hospital mortality rates for HNC. For the first time, representative population-based DRG data of 1,090,596 HNC cases treated between 2005 and 2018 were used to analyze in-hospital mortality trends for HNC in Germany. Overall, in-hospital mortality did not significantly decrease over time. There were relevant age disparities, which cannot be explained out of the data. Gender, tumor localization, and treatment types had influenced the mortality. Further studies with linkage to relevant influencing factors have to be performed.

## Figures and Tables

**Table 1:** Average in-hospital mortality rates for head and neck cancer in Germany for the years 2005-2018.

Parameter	Mean ± SD
All patients, age cohorts	
All ages	0.0434 ± 0.0183
35-49 years	0.0254 ± 0.0057
50-64 years	0.0329 ± 0.0034
65-79 years	0.0443 ± 0.0041
80+ years	0.0710 ± 0.0085
Male patients, age cohort	
All ages	0.0465 ± 0.0188
35-49 years	0.0287 ± 0.0038
50-64 years	0.0346 ± 0.0029
65-79 years	0.0470 ± 0.0033
80+ years	0.0757 ± 0.0072
Female patients, age cohort	
All ages	0.0403 ± 0.0173
35-49 years	0.0221 ± 0.0055
50-64 years	0.0311 ± 0.0031
65-79 years	0.0416 ± 0.0029
80+ years	0.0662 ± 0.0071
Localization	
Hypopharynx	0.0304 ± 0.0025
Larynx	0.0356 ± 0.0028
Oral cavity	0.0379 ± 0.0024
Nasopharynx	0.0391 ± 0.0033
Oropharynx	0.0512 ± 0.003
Salivary glands	0.0316 ± 0.0029
Treatment	
Biopsy (b)	0.0145 ± 0.0020
Surgery of the primary	0.0184 ± 0.0011
Neck dissection	0.0159 ± 0.0015
Radiotherapy	0.0207 ± 0.0028
Chemotherapy/antibodies	0.0118 ± 0.0012
No treatment	0.1047 ± 0.0026

SD: standard deviation.

**Table 2:** Negative binominal regression analysis of the influence of age and gender on the mortality between 2005 and 2018 in relation to the time.

Parameter	Relative risk	Lower 95% CI	Upper 95% CI	*p*
Age (years)				
35-49	1	Reference		
50-64	1.308	0.770	2.222	0.317
65-79	1.767	1.040	3.001	0.034
80+	2.826	1.663	4.803	<0.0001
Gender				
Male	1	Reference		
Female	0.856	0.590	1.243	0.413
Year	0.981	0.937	1.027	0.403

CI: confidence interval.

**Table 3:** Negative binominal regression analysis of the influence of the primary tumor localization on the mortality between 2005 and 2018 in relation to the time.

Parameter	Relative risk	Lower 95% CI	Upper 95% CI	*p*
Localization				
Hypopharynx	1	Reference		
Larynx	1.168	0.552	2.473	0.682
Oral cavity	1.242	0.587	2.629	0.567
Nasopharynx	1.284	0.606	2.722	0.509
Oropharynx	1.680	0.793	3.556	0.170
Salivary glands	1.035	0.489	2.192	0.928
Year	0.992	0.941	1.045	0.757

CI: confidence interval.

**Table 4:** Negative binominal regression analysis of the influence of the treatment type on the mortality between 2005 and 2018 in relation to the time.

Parameter	Relative risk	Lower 95% CI	Upper 95% CI	*p*
Treatment				
Biopsy	1	Reference		
Surgery of the primary	1.273	0.606	2.674	0.524
Neck dissection	1.099	0.522	2.312	0.804
Radiotherapy	1.430	0.681	3.003	0.345
Chemotherapy/antibodies	0.813	0.387	1.709	0.585
No tumor treatment	7.241	3.447	15.211	<0.001
Year	0.993	0.942	1.046	0.786

CI: confidence interval.

## Data Availability

The data sets used during the current study are available from the corresponding author upon reasonable request.
